# Action of Homœopathic Globules†These notes on Dr. Malin’s book were communicated (in 1864) by the late Dr. John Epps, and are sent us by Dr. Berridge. We republish them for the benefit of our Milwaukee friends, who can read them in the leisure hours not devoted to Jaeger.—Ed.

**Published:** 1881-05

**Authors:** 


### THE ACTION OF HOMŒOPATHIC GLOBULES ON PERSONS IN HEALTH.†

Dr. Malin, in a pamplet entitled, "How can such Small Doses have any Effect?" makes the following statement:

"If a man in health takes for some consecutive days any one of the homœopathic medicines, it will soon produce symptoms similar to those which it cures, and as the evident result of its action, an artificial disease will be created.

"While I was studying homœopathy under Hahnemann's teaching at Paris, I used to make trial on myself of the homœ-

† These notes on Dr. Malin's book were communicated (in 1864) by the late Dr. John Epps, and are sent us by Dr. Berridge. We republish them for the benefit of our Milwaukee friends, who can read them in the leisure hours not devoted to Jaeger.—Ed.

opathic remedies, in order to ascertain if really and truly they were invested with any medicinial power, and to what extent.

"I had received from a well known homœopathic practitioner a few globules, carefully folded up in small paper cases, each case accurately numbered, and the numbers corresponding to the names of the remedies sealed up apart. Being in good health, and in the practice and enjoyment of the most regular habits, I took, for several consecutive mornings, a few globules out of one of the said paper cases, without altering my deit, and following, as usual, my daily studies and occupations.

"After some days, however, I was seized with many unusual symptoms; great sleepiness, fatigue, shivering, soon followed by fever; moreover, a singular eruption of transparent vesicles, on an inflamed ground, accompanied with great sense of burning and itching, soon spread from head to foot. Anxious and alarmed, my friends had begged the attendance of two of the first allopathic physicians of Paris, who daily visited me, prescribed for me, and were quite at a loss as to the nature and name of this new sort of eruptive fever. Hardly recovered from the most acute stage, I stated all the circumstances to Hahnemann, and was not a little surprised to hear him say: 'You have taken too great a dose of some homœopathic medicine.... and it must be *Rhus toxicodendron*'.... But my surprise became greater still when, by referring to the names sealed up and corresponding to the numbers on the paper cases, we found that Hahnemann was right; I had been taking a daily repeated dose of a few globules of *Rhus toxicodendron*, and its antidote now rapidly removed the remaining symptoms.

"At a later period, while at Lyons, and pursuing the study and investigation of homœopathy, I continued the personal experiment of homœopathic medicines. Under the same circumstances, and in a similar way as stated above, I took daily some globules, of a remedy, to me entirely unknown, till after a few days of trial, I was seized with giddiness, nausea, a violent oppressive frontal headache, depression of spirits, continued want of appetite, and many other symptoms which, in spite of much care and attention, persisted day after day, and obliged me to give up all study and occupation. While I was in that state, a homœopathic physician chanced to call, and was surprised to find me laid up ill, and suffering. He stated at once that I had taken *Nux vomica*, and gave me the antidote to it. Here again the correctness of the assertion was proved by the result of the treatment, and by a reference to the names corresponding to the numbers on the packets.

"Such proofs of the action of homœopathic medicines, so palpable and so striking, can not fail to support our statement, that they are, in homœopathic doses, active and powerful."

It may be added that the small doses referred to are globules, thus described:

"For practical use, small globules, made of pure sugar of milk and gum, are soaked in the liquid preparations; acting like so many small sponges, they become impregnated with them, and are carefully dried and preserved in phials. These globules are thus the bearers of the medicaments, and they are prescribed either by themselves or mixed with pounded sugar of milk or dissolved in water, according as the case may require."

Dr. Malin adds—

"At first Hahnemann used the remedies prepared in the old way, but, gradually experience taught him the present superior manner of preparing them. He therefore adopted it, and it is now employed by all homœopathists."

